# Recent advances in porous microneedles: materials, fabrication, and transdermal applications

**DOI:** 10.1007/s13346-021-01045-x

**Published:** 2021-08-20

**Authors:** Leilei Bao, Jongho Park, Gwenaël Bonfante, Beomjoon Kim

**Affiliations:** 1 Institute of Industrial Science, The University of Tokyo, Tokyo, Japan; 2 LIMMS/CNRS-IIS UMI 2820, The University of Tokyo, Tokyo, Japan

## Abstract

In the past two decades, microneedles (MNs), as a painless and simple drug delivery system, have received increasing attention for various biomedical applications such as transdermal drug delivery, interstitial fluid (ISF) extraction, and biosensing. Among the various types of MNs, porous MNs have been recently researched owing to their distinctive and unique characteristics, where porous structures inside MNs with continuous nano- or micro-sized pores can transport drugs or biofluids by capillary action. In addition, a wide range of materials, including non-polymers and polymers, were researched and used to form the porous structures of porous MNs. Adjustable porosity by different fabrication methods enables the achievement of sufficient mechanical strength by optimising fluid flows inside MNs. Moreover, biocompatible porous MNs integrated with biosensors can offer portable detection and rapid measurement of biomarkers in a minimally invasive manner. This review focuses on several aspects of current porous MN technology, including material selection, fabrication processes, biomedical applications, primarily covering transdermal drug delivery, ISF extraction, and biosensing, along with future prospects as well as challenges.

**Graphical abstract:**

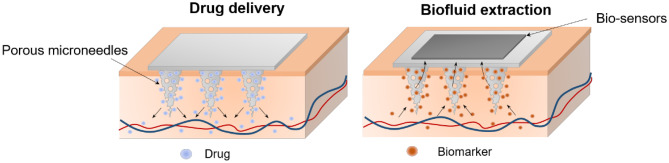

## Introduction

**Fig. 1:**
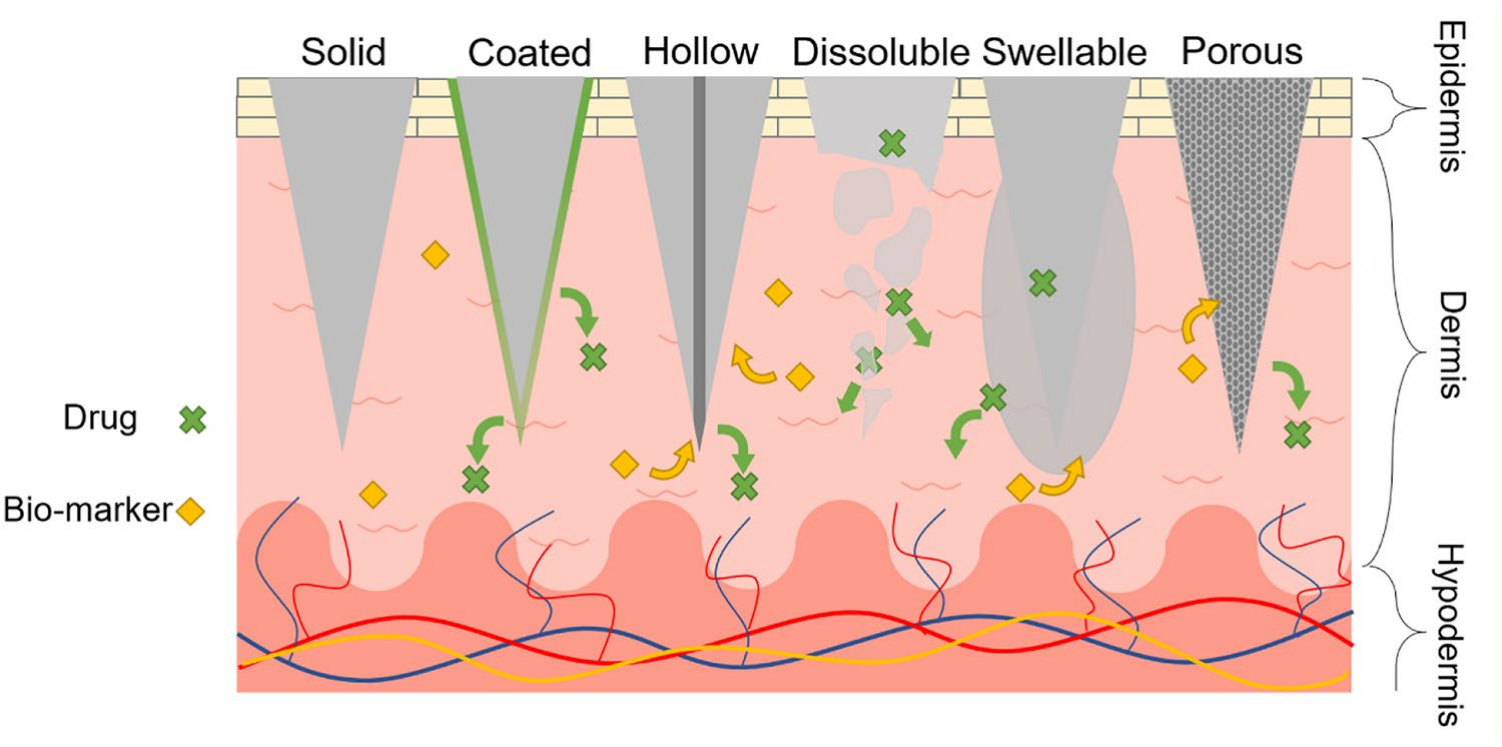
Different structural and functional microneedles (MNs) for painless drug delivery or biosensing

Microneedles (MNs), having microscopic needle structures, were initially developed to facilitate transdermal drug delivery by piercing human skin and providing transport conduits across the stratum corneum with a thickness of 10–15 µm [1, 2]. MNs have received significant attention in recent years owing to their micro-sized structure that can penetrate the skin painlessly without stimulating nerve endings, which results in minimal invasiveness and better patient compliance [3–5]. Taking advantage of its microscopic structures, MNs have been developed as a suitable tool for painless drug delivery and biosensing. According to the matrix material and structure, MNs are divided into six categories, as depicted in Fig. 1: solid, coated, hollow, dissoluble, swellable, and porous MNs.

The first MNs, which aimed at enhancing the skin permeability for transdermal drug delivery, were of the solid type [1]. Solid MNs are generally fabricated using silicon, metal, or polymer materials, which have sufficient mechanical strength to puncture the stratum corneum and penetrate the epidermis of human skin [6–11]. After the penetration and removal of solid MNs from the skin, drug components are transported into the dermis layer through pathways formed by the MNs. Because of these pathways, the skin permeability can be enhanced to facilitate transdermal delivery of both micro- and macro-molecular (over 600 Da) drug material. However, accurate control of the drug amount is difficult because of the gradual closure of formed pores over time. Moreover, the leftovers of broken solid MNs, which are manufactured from metals, silicon, and non-biocompatible polymers, are considered harmful to the human body because they cannot be biodegraded.

Conversely, the development of coated MNs overcame the issue of pore closure after the penetration and removal of solid MNs. By coating the drug material on the surface of the MNs, the drug can diffuse into the skin directly while maintaining the pathways formed by the penetration without leaving drug waste on the surface of the skin. Similar to the solid type, the coated MNs are fabricated using silicon, metals, or polymers [12–14]. Coated MNs have the advantages of achieving controllable drug dose by adjusting the amount of coated drug. In addition, drug stability can be achieved in the long term owing to the solid phase of the coated drug material. However, the amount of drug material is significantly restricted because of the limited surface area of micro-scaled MN structures.

Hollow MNs, which are manufactured using silicon, metals, or non-dissolving polymers, enable the administration of a large dose of the drug [15–19]. One among the several advantages of hollow MNs is that the flow rate of the drug solution into the skin layer is controlled by adjusting the infusion pressure as well as the insertion depth. In addition, interstitial fluid (ISF) inside the skin layer can be extracted by capillary action owing to its hollow structures. Conversely, the mechanical strength of hollow MNs is relatively lower than that of solid or coated MNs.

Dissoluble MNs are generally made of biocompatible and biodegradable polymers or sugar-encapsulating active substances [20–23]. Thus, MNs dissolve when inserted into the skin, releasing the loaded drug into the skin. Here, the release kinetics of a drug usually depends on the constituent of the matrix material. One noteworthy feature of dissoluble MNs is that the risk of leaving leftovers or structural wastes inside the skin layer is decreased by using water-soluble MN materials.

Swellable MNs are generally fabricated using physical crosslinking techniques such as exposure to ultraviolet (UV) light with photoinitiators [24]. Water-soluble swellable MNs with encapsulated drugs could achieve prolonged and uniform drug release with adjustable delivery rates by altering the crosslinking degree. In addition, ISF can be extracted in a minimally invasive manner and stored in the MNs using swelling properties [25–28]. Furthermore, the mechanical strength and swelling ability can be adjusted by tuning the concentration of the prepolymer as well as the crosslinking time. Currently, the biomarkers in ISF are mainly detected and analysed after the targeted ones are recovered from the swollen MNs by post-processing such as centrifugation and solvent extraction [26], and hydrogel MNs have great potential in the integration with lab-on-chip biosensing devices and detect the targeted biomarker directly and real-timely.

Recently, porous MNs, which have interconnected micro-sized pores throughout the entire structure of MN, have attracted significant attention [29–34]. Porous MNs are generally created from biocompatible metals, ceramics, or polymers with small randomly distributed and interconnected pores. By taking advantage of continuous pores, the drug solution can be absorbed spontaneously by capillary action with an active pharmaceutical ingredient (API) being stored or maintained in a dried form for additional minimally invasive drug delivery. In addition, the rapid absorption and collection of ISF can be achieved using porous MNs through capillary action. Thus, ISF extraction and the subsequent analysis of biomarkers in fluids can be achieved using porous MNs integrated with lab-on-chip biosensing devices. Meanwhile, a trade-off between the porosity and the mechanical strength is necessary owing to the large volume of voids inside the MN when compared to solid MNs, which results in an increase in fragility.

From the application perspective, transdermal drug delivery utilising MNs has been widely researched. Drug materials such as insulin [35–38], anti-cancer agents [39], hormones [40], deoxyribonucleic acids [41], anti-obesity agents [42], and drug-loaded nanoparticles [43] are administered using the different types of MNs described above for minimally invasive transdermal drug delivery with a rapid release. In addition, vaccine delivery through transdermal pathways has attracted significant attention as a new application for drug delivery using MNs. For example, dissoluble MNs incorporating vaccine materials especially showed their potential in MN-based vaccine delivery [20, 44].

For further applications using MNs, the extraction of body fluids, including blood and ISF, has recently emerged as an alternative to conventional hypodermic needles for painless extraction, followed by monitoring as well as diagnoses. Among them, the skin ISF, which is primarily located in the epidermis and dermis layers of the human skin, was reported to have abundant biomarkers such as glucose, alcohol, lactate, cortisol, cholesterol, and other proteins [45]. Several studies related to ISF extraction and monitoring were performed using MNs, which include the solid, hollow, swellable, and porous types of MNs. Solid-type MNs were used to collect dermal ISF by piercing the skin followed by absorption using a paper strip [46]. Hollow MNs were employed for rapid ISF extraction by capillary action owing to their structure. They were also integrated with biosensors or microfluidic channels to achieve the transportation of collected fluids and the subsequent analysis [47–51]. However, it should be noted that both solid- and hollow-type MNs were fabricated using non-biocompatible materials, which results in potential risks to the health of patients with long-term administration or broken leftovers in the skin [49–51]. Swellable MNs, generally created from crosslinked hydrogels, enable the continuous absorption of body fluids until saturated [27, 52–57]. Such hydrogels fabricated using biocompatible materials can be employed for safe ISF extraction and the detection of various biomarkers, including glucose, cholesterol, and lactate. However, a post-processing step is usually necessary to extract the collected ISF from the polymeric crosslinking network of MNs for further analysis.

Porous MNs were developed only 5 years ago for collecting skin ISF. Porous MNs have interconnected micron-sized pores through which fluids can be transported because of the channel-like connected pores by capillary action, thereby transporting the collected fluid to the analysis system directly. Moreover, a wide range of biocompatible materials have been used for the fabrication of porous structures to extract skin ISF in a passive manner, enabling the collection of body fluids by detecting and analysing biomarkers using biosensors [34, 58]. Although porous MNs have been researched for their various applications as well as unique characteristics, a detailed review on porous MNs has not been performed yet, as determined through our investigations so far. In this review paper, we reviewed porous MNs in terms of base materials and fabrication methods. In addition, we introduced biomedical applications using porous MNs and discussed their current challenges as well as future prospects.

## Materials and fabrication of porous microneedles (MNs)

### Materials

Both non-polymeric and polymeric materials can be used for the fabrication of porous structures. In this chapter, the material properties, representative fabrication methods, biomedical applications, and characteristics of porous structures are described with respect to each material and listed in Table 1.

**Table 1:** Materials, fabrication methods, pore characteristics, and applications of porous microneedles (MNs)

Material	Fabrication method	Applications	Pros	Cons	Porous structure
**Non-polymer**					
Silicon	Electrochemical anodization [61, 62]	Drug delivery	- Biocompatibility and biodegradability of porous silicon	- Bulky equipment and clean room required - Limited needle shapes	[61]
Alumina	Sintering process [31, 66–69] Electrochemical anodization [65]	Drug delivery [31, 66–68] ISF sampling [66]	- Strong mechanical properties - Controllable porosity	- Not biodegradable, brittle - Heating process at high temperature conditions [31, 66–69]	[66]
CaS & CaP (bioceramics)	Mild micro-moulding [71]	Drug delivery	- Drug added into the bioceramics before moulding - Flexibility in drug loading	- Variance in the drug release due to the water solubility of CaS and CaP	[71]
Titanium	Wet etching [77] Sintering process [30]	Drug delivery	- Biocompatible metal - Easy penetration into skin	- Microfabrication with clean room environment [77] - Complex sintering process [30]	[30]
Stainless steel (316L)	Sintering process [33]	Drug delivery / ISF sampling	- Biodegradable metal - Ability to store dehydrated drugs and extract biofluids	- Complex fabrication comprising of hot embossing, debinding and sintering - Thick and rigid substrate	[33]
**Polymer**					
Poly (ethylene glycol-co-methacrylic acid)	Phase separation [81]	Drug delivery / ISF sampling	- Controllable pore size and mechanical strength	- Difficulty in filling the MN mould due to the viscous polymer solution - Toxic and harmful organic porogen	[81]
Poly (lactic-co-glycolic acid) (PLGA)	Emulsion and coating [32] Hot embossing [38] Porogen leaching [58]	Drug delivery [32, 38] ISF sampling [58]	- Biocompatible and biodegradable material - Tuneable porosity by modulating porogen amount [32, 58]	- Limitation of the drug dosage [38] - Long time to remove the porogen [58]	[58]
Polydimethylsiloxane (PDMS)	Porogen leaching [34, 91]	ISF sampling	- Controllable porosity by tuning porogen ratio	- Long time to remove the porogen - Weak mechanical strength - Manual compression required to drive ISF flow	[34]
Cellulose acetate (CA)	Phase inversion [92]	Drug delivery / ISF sampling	- Tuneable porosity and mechanical strength - Phase inversion is simple and versatile for many types of polymers	- The organic solvent (e.g. dimethyl sulfoxide is toxic and harmful to the human skin)	[92]
Polylactic acid (PLA)	Emulsion and bonding [96, 97]	ISF sampling	- Biocompatible and biodegradable material - Suitable for mass MNs production	- Mechanical strength needs improved - Requirement of complete evaporation of the organic solvent	[96]

#### Non-polymers

##### Silicon

Initially, MNs were fabricated from silicon material by using conventional microelectromechanical system (MEMS) technologies. Solid, coated, and hollow MNs with various dimensions and aspect ratios were fabricated using a deep reactive ion etching (DRIE) process [15, 59, 60]. However, the MNs are prone to break and remain in the human skin, which may cause an infection because of the fragility of silicon. To overcome such problems, biodegradable nanostructured porous silicon was fabricated on the tip of MNs [61, 62]. After the fabrication of the silicon MN array by the DRIE process, electrochemical etching was performed using an electrolyte containing a mixture of acetonitrile (MeCN) and diluted hydrofluoric acid (HF) to generate porous silicon structures. The Young’s modulus and yield strength of the fabricated porous silicon were 2.4 GPa and 100 MPa, respectively. The MNs with porous silicon tips showed enhanced drug delivery and increased skin permeability of approximately 5–6 times when compared to the passive transdermal delivery without MNs. Owing to the biocompatibility and biodegradability of porous silicon, it is biodegraded within several weeks even if the tips break off and remain inside the skin [63]. Moreover, porous structures are formed only in the tip part, which ensures sufficient mechanical strength for successful penetration into the skin. However, there are certain disadvantages in using silicon materials for MNs. For example, processes for silicon materials require complicated MEMS-based processes as well as a clean room environment. Furthermore, the shapes of MNs are strictly limited by the material properties of silicon substrates and the fabrication process.

##### Bio-ceramics

Alumina is a first-generation bio-ceramic material and has been applied in biomedical and clinical applications such as implants owing to its biocompatibility and high mechanical strength [64]. Recently, alumina has been extensively employed to fabricate porous MNs with micro- or nano-sized interconnected pores for fluid transportation. Alumina-based porous MNs can be prepared by sintering or aluminium anodization [31, 65–69]. For the sintering method, alumina particles and binders are used for forming pores directly at high-temperature conditions (e.g. 1200–1500 °C). Here, the particle size was adjusted to be sufficiently small to fill the cavities of the female mould and to be sufficiently large to form the desirable pore size. Consequently, porous alumina structures with pore diameters ranging from 15 nm to 1.5 µm and porosity ranging from 22 to 60% have been reported. Moreover, the fracture force of a single needle, measured to be 3.1 ± 0.32 N, was sufficient for penetration into the skin of a rat [66].

While considering the applications, alumina-based porous MNs are used to store and deliver antibodies [31], insulin [66], peptide vaccines [67], and nanoparticles [68] as well as the extraction of ISF for further detection of biomarkers (e.g. glucose), which was achieved within 15 min [66].

The benefits of using porous alumina include strong mechanical properties when compared to polymer MNs and improved controllability of porosity. In the case of the sintering process, porosity can be controlled by adjusting the sintering temperature. Similarly, the pore diameter and density can be modified by changing the applied voltage during the chemical etching process. Conversely, one of the limitations of using alumina is that it is brittle and can be easily broken inside the human body. Although such porous structures (e.g. silicon and alumina) are biocompatible, their brittleness is considered to be undesirable in biomedical and clinical devices.

Meanwhile, other bio-ceramics such as calcium sulphate dihydrate (CaS) and calcium phosphate dihydrate (CaP) have also been applied to fabricate porous MNs as bone substitute materials because of their superior biocompatibility [70, 71]. Porous CaS and CaP MNs can be fabricated using a simple micro-moulding process, and the details are described in the “Fabrication methods” section. While considering the mechanical strength, it was reported that the average compressive strengths of CaS and CaP were measured to be 25.7 and 24.8 MPa, respectively, while the hardness was reported as 951.3 MPa for CaS and 872.8 MPa for CaP. For biomedical applications, it was reported that porous MNs fabricated using CaS and CaP were employed for drug loading and controlled release.

##### Metals

Metals such as titanium and stainless steel can be used to fabricate porous MNs. Among several types of metals, titanium has been extensively applied for biomedical devices such as dental and orthopaedic implants owing to its biocompatibility and excellent mechanical strength [72]. By capitalising on these properties, solid [73], coated [74], hollow [75, 76], and the recently developed porous titanium MNs were researched for various biomedical transdermal applications such as macro-molecular drug (e.g. insulin) loading and delivery [30, 77]. Microfabrication using wire-electrode cutting and wet etching was used to fabricate porous titanium MNs using a porous titanium wafer substrate that had numerous connected internal micro-holes. Similar to alumina, porous titanium MNs can also be fabricated using a micro-moulding and sintering method. The porosity and pore diameter of the titanium MNs were obtained as 30.1% and 1.3 µm, respectively, and compression force to pierce the human skin was measured as 25 mN/needle [30].

Similar to the titanium material, medical grade stainless steel (316L) has been used for MNs for over several decades owing to its biocompatibility under increased clinical applications and acceptance of the patients [33, 78–80]. Stainless-steel-based porous metallic MNs (PMMNs) are fabricated by hot embossing, debinding, and sintering processes using stainless steel powder, pore fillers, and binders. The total internal porosity of the PMMNs was measured to be 36% with a pore diameter of 2.2 µm, enabling the storage of dehydrated micro- and macro-molecular drugs or the absorption of biological fluid. In addition, it was reported that the PMMN array possessed sufficient mechanical strength to pierce porcine skin. Thus, in summary, MNs manufactured from silicon, bio-ceramics, and metal materials have better mechanical properties, which results in the penetration of the skin without any fracture when compared to the polymeric MNs described in the next section. However, while considering the applications targeting human skin, the substrate as well as the matrix material of the metallic MN patch is too thick or hard and lacks sufficient flexibility to achieve conformal contact onto the human skin.

#### Polymers

Polymers have received considerable attention because they have unique mechanical characteristics such as the ability to withstand large bending forces without being fractured. More importantly, polymeric MNs have advantages over other materials as they can be easily fabricated without complicated micromachining processes as well as equipment and the requirement for a clean room environment. In the last decade, several types of polymers have been researched and developed as materials for porous MNs for various biomedical applications such as transdermal drug delivery and the extraction of biofluids.

##### Poly (ethylene glycol-co-methacrylic acid)

Synthesised polymeric materials such as poly (ethylene glycol-co-methacrylic acid) were developed to produce porous structures by using bulk polymerisation techniques with different porogens to achieve various fluidic characteristics and mechanical properties [81]. Poly (ethylene glycol) diacrylate and trimethylolpropane trimethacrylate (TRIM) as biocompatible crosslinking agents and methacrylic acid as a nontoxic functional monomer were used to synthesise poly (ethylene glycol-co-methacrylic acid), along with toluene, ethyl acetate, or diethyl ether as porogenic solvents for the formation of nano- and micro-sized pores [81–85]. Both pore sizes and mechanical strength were controllable by modulating the amount of crosslinkers and functional monomers as well as the type of the porogen. Here, porous structures with pore sizes ranging from 0.7 nm to 5 µm were achieved. Moreover, in terms of the mechanical properties using different porogenic solvent groups, 1.91–139.35 MPa of average maximum strength and 0.31–0.66 of average maximum strain were attained, while various flow fluid characteristics were achieved. Owing to the tuneable flow fluid rate obtained by using different porogens, the porous MN array has significant potential for controlled drug release or biofluid transport. However, organic porogens (e.g. toluene, ethyl acetate, or diethyl ether) are toxic and harmful to the human body, which require complete removal from MNs.

##### Poly (glycidyl methacrylate)

Similar to poly (ethylene glycol-co-methacrylic acid), poly (glycidyl methacrylate) (PGMA) synthesised by photo-polymerisation in the presence of a porogenic solvent was employed to produce porous structures. Porous PGMA MNs are fabricated by using the monomer, glycidyl methacrylate (GMA), and crosslinkers, TRIM and triethylene glycol dimethacrylate (TEGDMA), mixed with poly (ethylene glycol) (PEG) solution as a porogen. After the MNs are produced by photo-polymerisation, micropores are formed by a leaching process to remove the PEG porogens [29, 86]. A pore diameter of 1 µm on average is achieved. In the evaluation of penetration using the PGMA porous MNs applied with a vertical force of 0.5 N/needle, more than 80% of the penetration efficiency was achieved with a porosity below 50%. However, it should be noted that methanol must be removed completely because it may be harmful to the human body if it remains inside the MNs. For its applications, the developed porous PGMA MN array was reported to be versatile in both drug delivery and ISF sampling. In addition, the porous PGMA MN array, which is conductive, was integrated with electrodes for transdermal local monitoring of intercellular swelling by measuring the DC current of the epidermis layer of the skin.

##### Poly (lactic-co-glycolic acid)

Poly (lactic-co-glycolic acid) (PLGA) is a copolymer of poly (lactic acid) (PLA) and poly (glycolic acid) (PGA). For nearly three decades, it has been extensively employed in the field of MNs for biomedical and clinical applications (e.g. drug-encapsulated nano- and micro-particle delivery) because of its biocompatibility and biodegradability [87–89]. Several approaches, such as emulsion followed by coating, hot embossing, and porogen leaching were developed to fabricate PLGA porous structures [32, 38, 58]. Using these methods, porosity is controllable and adjustable, resulting in pore diameters ranging from 1.9 to 15 µm; moreover, a porosity of 20.1 to 63% can be achieved. By far, porous PLGA MNs have been applied in drug delivery (e.g. insulin) and biological fluid extraction as well as subsequent analysis.

##### Polydimethylsiloxane

Polydimethylsiloxane (PDMS) is a well-known polymeric material used in the field of microdevices, especially as biomedical devices, owing to its notable chemical inertness, processability, flexibility, and biocompatibility [90]. As PDMS is in a liquid state at room temperature, porogens such as micro-sized salt particles can be mixed easily and homogeneously by conventional mixing methods. PDMS porous structures are then fabricated using micro-moulding, curing, and leaching processes [34, 91]. Unlike other polymeric MNs, the fabricated porous structure is similar to a sponge with low Young’s modulus of approximately 3.7 kPa, in the case of 80% porosity, which results in difficulty in penetrating the human skin. Consequently, the MN structure is coated with hyaluronic acid (HA) to improve the mechanical strength, resulting in 0.34 N/needle of buckling force. The results showed that a porosity of 60% is optimal for ISF extraction. In addition, the fabricated porous PDMS MN can be integrated with a microfluidic chip to achieve ISF collection as well as sensing of the glucose level, although manual compression action is required to drive ISF flow because of its sponge-like structures.

##### Cellulose acetate

Cellulose acetate is a synthetic polymer that is also regarded as a proper base material to fabricate MNs owing to its biocompatible characteristics [92]. The porous structures inside the MNs are fabricated by dissolving cellulose acetate in dimethyl sulfoxide (DMSO), casting using an MN female mould, and finally immersing the entire mould in water to induce phase inversion. Here, by using phase inversion, the polymer solution can be transformed from a liquid to a solid state after being immersed in a non-solvent bath, which results in pore formation from the solidified polymers [93]. By adjusting the concentration of cellulose acetate in DMSO, an organic solvent with a porosity of 40–90% and Young’ s modulus of 2.5–40 MPa can be achieved. In addition, it was reported that biofluid extraction and insulin delivery were both achieved using continuous pores formed inside porous cellulose acetate MNs. However, the organic solvent (e.g. DMSO) remaining in the solidified MNs is considered to be harmful to the human skin [94].

##### Poly (lactic acid)

As a widely used biocompatible and biodegradable polymer, PLA is extensively employed in the field of biomedical applications such as drug delivery using MNs [35, 93–95]. In particular, PLA microparticles have been researched and developed to encapsulate APIs for sustained release in the human body. Conversely, PLA microparticles can be used to fill the MN mould and form interconnected voids between each micro-particle [96, 97]. Basically, the emulsion method, which mixes PLA solution and surfactant, is used to yield PLA microparticles with a size range of 1–30 µm. The prepared PLA microparticles can be cast onto the MN mould and applied with ultrasonic welding or heat treatment to bond the microparticles, resulting in interconnected micropores. The overall porosity of PLA was determined to be 75%, intended for biofluid extraction and further analysis; however, the mechanical strength requires reinforcement and optimisation. Although PLA is a biodegradable polymer, it has a high melting point of approximately 170 °C; thus, more energy must be applied during the heating treatment process. In addition, the toxic organic solvent used in the emulsion process must be evaporated completely to ensure that no harmful substances remain in the MNs.

### Fabrication methods

In this chapter, current fabrication methods for porous structures as well as porous MNs are described. In addition, previous literature pertaining to each method are reviewed and discussed in detail.

#### Electrochemical anodization

**Fig. 2:**
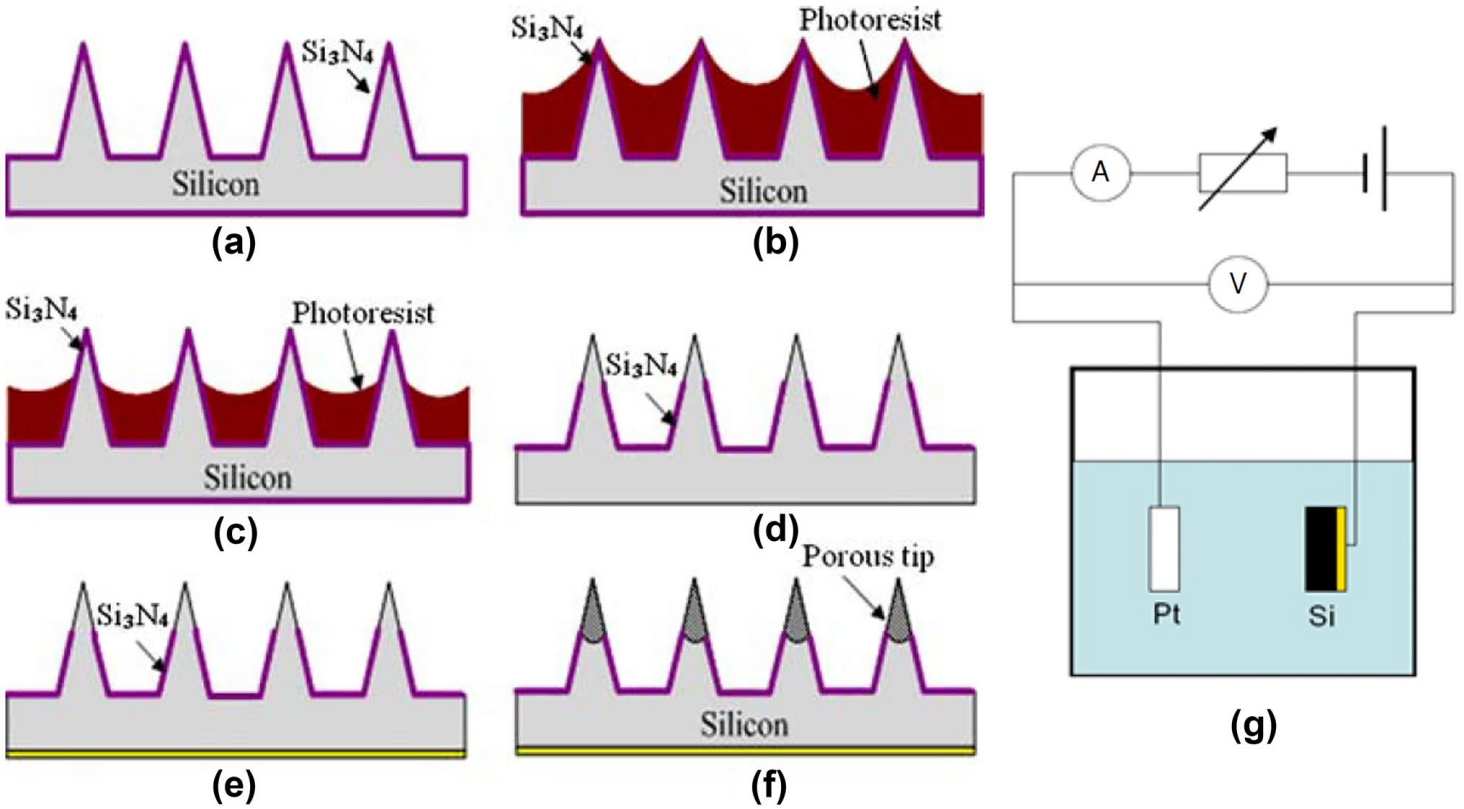
Fabrication process of the porous structures on the silicon MNs: **a** deposition of silicon nitride layer on silicon MN array by low-pressure chemical vapour deposition; **b** coating of photoresist; **c** photoresist thinning by reflowing; **d** removal of photoresist by reactive ion etching using O_2_; **e** deposition of a thin gold layer on the backside of silicon MN array as conductive layer; **f** porous silicon tips formed by electrochemical etching; **g** experiment setup for anodic electrochemical etching (reproduced with permission [62])

For the formation of porous silicon structures, electrochemical anodization process is commonly used owing to its accurate replicability and easy modification. Silicon MN arrays are prepared by employing microfabrication processes based on MEMS technologies [59, 61, 62]. After silicon MNs are fabricated, a silicon nitride layer is deposited on the MN surface using low-pressure chemical vapour deposition method (Fig. 2a). Subsequently, the photoresist resin is spin coated and annealed (Fig. 2b). Here, because of the reflowing, the photo-resistance becomes thinner on the tips of the MNs. By using oxygen reactive ion etching, the resin left on the tips is removed (Fig. 2d). Finally, porous silicon is generated by forming backside electrodes (Fig. 2e) and anodic electrochemical etching using a mixture of MeCN and HF (Fig. 2g). One of the advantages of electrochemical methods is that the pore size and porosity are tuneable by adjusting electrochemical etching parameters such as the concentration of HF, current density, and anodic time.

Similar to silicon-based porous MNs, a porous-structured anodised aluminium oxide (AAO) MN array can also be fabricated by an electrochemical anodization process [65]. First, the aluminium micropillar array is prepared by a micromachining process and long-time electropolishing. Subsequently, a nanostructured porous AAO surface is formed by anodising in H_3_PO_4_, where a platinum plate is used as the cathode. Here, with the positive potential applied to aluminium in the H_3_PO_4_ electrolyte, aluminium is oxidised to Al^3+^; then, it reacts with O^2−^ to form aluminium oxide, which is a porous structure. Similarly, the pore size is controllable, ranging from 15 to 500 nm, by adjusting the concentration of the electrolyte, temperature, and anodization time.

#### Wet etching

Isotropic wet etching can be used to fabricate porous titanium MNs [76]. In this case, a porous titanium wafer is directly employed as a substrate for the formation of porous structures. After the wafer is cut into one patch containing microcolumns by wire-electrode cutting, isotropic wet etching using a mixture of acids (HNO_3_: HF: H_2_O = 5: 2: 7) is applied to etch each microcolumn into the tapered structures. In this process, continuous pores are first blocked by metal chips or dust during the cutting process. However, the pores are finally connected after the etching process using acids that eliminate chips and dust.

#### Mild micro-moulding

**Fig. 3:**
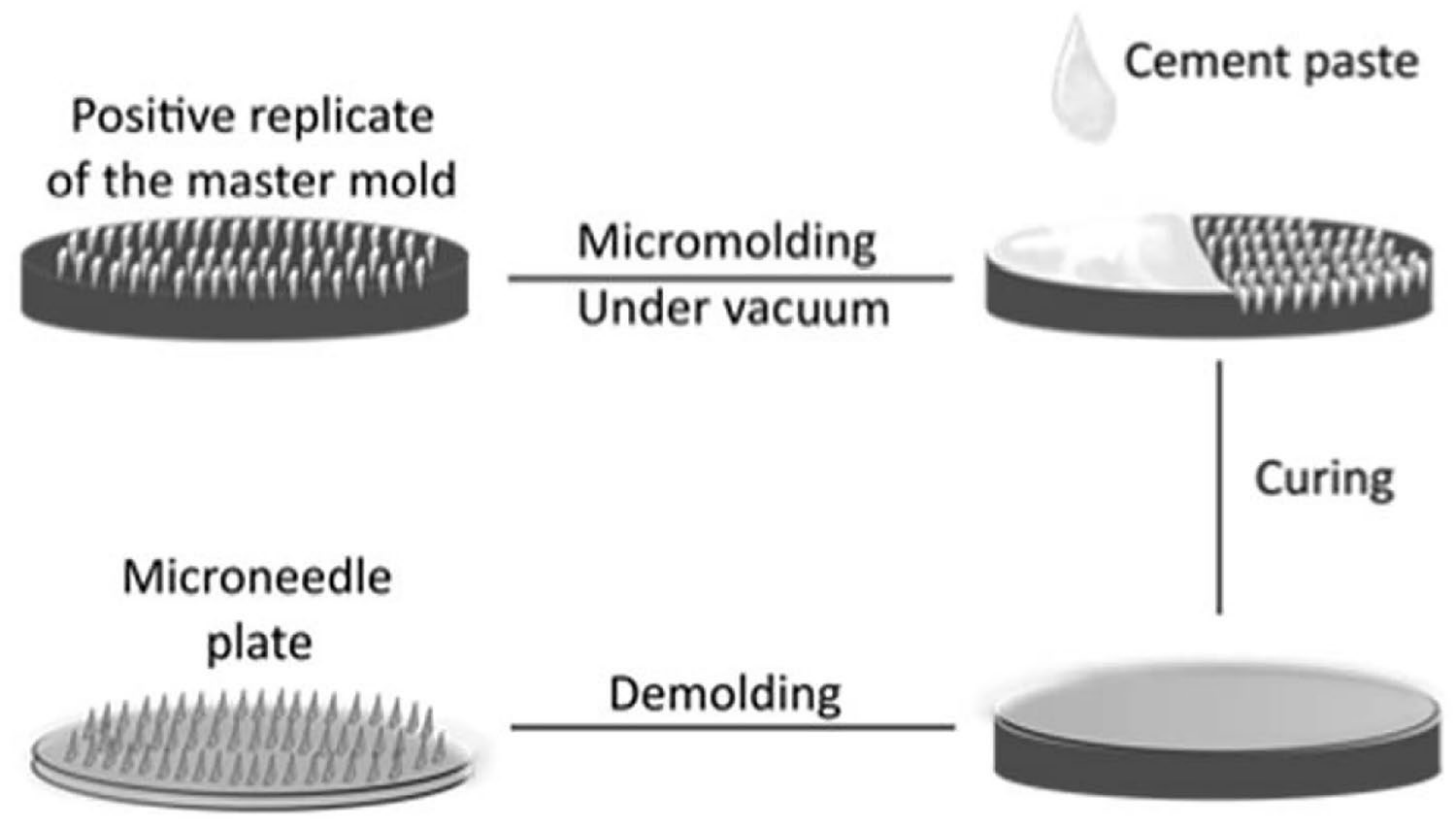
Schematic diagram of the fabrication process for porous calcium sulphate dihydrate and calcium phosphate dihydrate MNs. The blended ceramic paste was cast onto the MN mould and filled into the cavities under vacuum. Then, the entire mould was cured and demoulded (reproduced with permission [71])

Porous MNs using bio-ceramic materials such as CaS or CaP can be produced by a mild micro-moulding approach [71]. In this study, CaP porous structures were prepared by blending 45 wt% Ca_3_(PO_4_)_2_, 55 wt% Ca(H_2_PO_4_)_2_·H_2_O, and 0.5 M citric acid solution in 0.4 liquid/powder ratio. Subsequently, the mixed materials were poured, vacuumed, and cured in a PDMS female mould at 37 °C for 48 h (Fig. 3f). In contrast, CaS MNs with interconnected pores were produced by mixing CaSO_4_·0.5H_2_O with water in a liquid/powder ratio of 0.4 and curing at ambient conditions. Using this mild micro-moulding method, drug can be directly blended into the bio-ceramic paste and loaded into the MN female mould together. The mild drying conditions would preserve the APIs and the drug loading can be adjusted with flexibility.

#### Sintering process

For the formation of porous structures, the sintering process has been employed on metallic powder or particles to fuse them together and directly form interconnected pores.

**Fig. 4:**
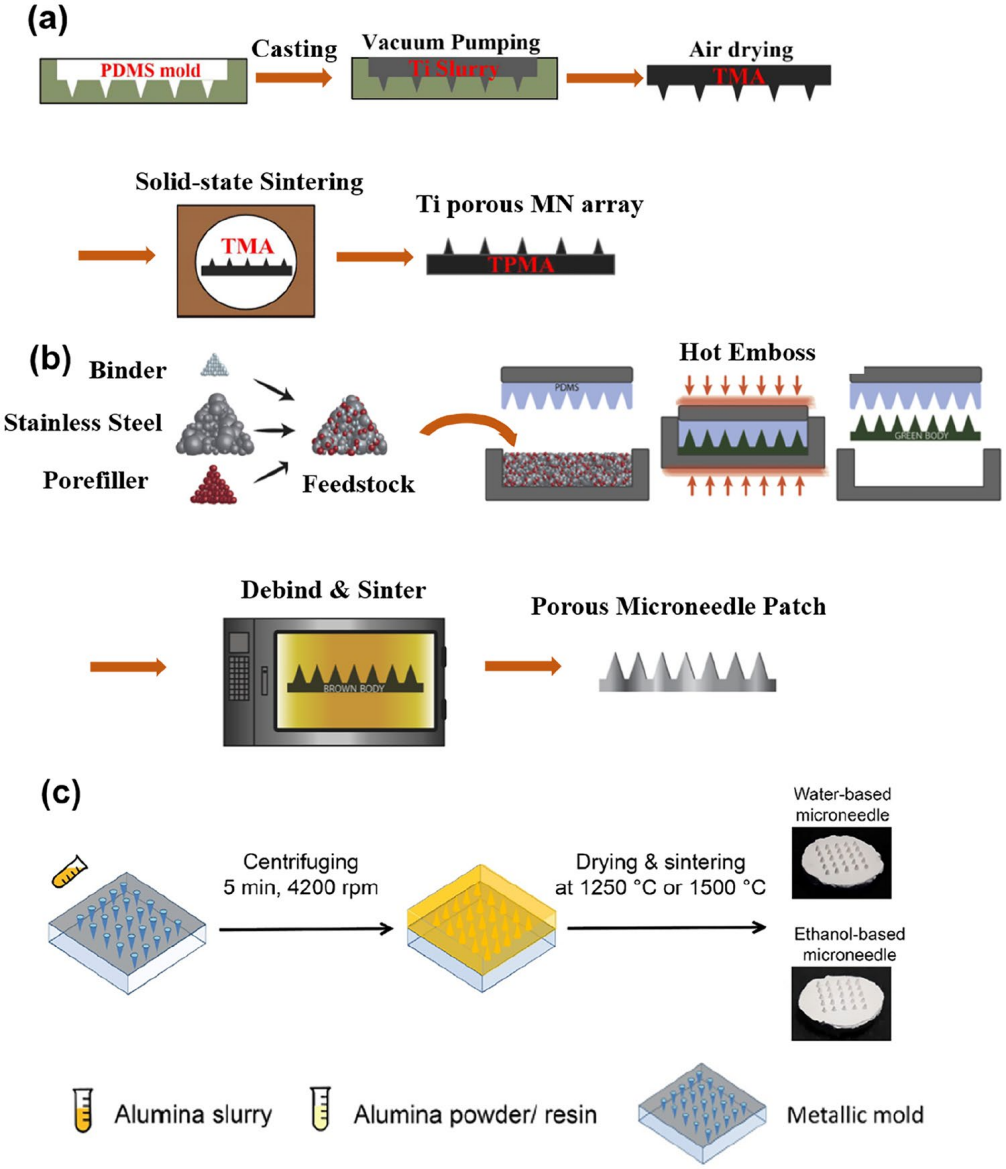
Sintering process to fabricate porous structures: **a** titanium MNs (reproduced from [30]); **b** stainless steel MNs (reproduced with permission [33]); **c** alumina MNs (reproduced with permission [66])

A porous titanium MN array was fabricated by combining metal injection moulding and sintering processes as depicted in Fig. 4a [30]. First, a slurry containing titanium, ethanol, poly (vinyl butyral) (PVB) as a binder, butyl benzyl phthalate as a plasticiser, and Solsperse as a dispersant were prepared for casting onto a PDMS female mould. Vibration by ultrasound and removal of the air bubbles by 15 min of vacuum pumping were performed to allow the titanium slurry to fill the cavities of the PDMS mould. Subsequently, the green body of the titanium MN array was air-dried at room temperature for 48 h and peeled off. During the sintering process, the binder, plasticiser, and dispersant were decomposed, and the titanium MN array was sintered to fuse and bond the titanium nanoparticles together. A robust interconnected porous structure could be produced owing to the high-temperature bonding process. The fabricated porous titanium MN had cone-shaped sharp tips with an average pore diameter and porosity of 1.3 µm and 30.1%, respectively.

Similar to porous titanium MNs, porous stainless steel MNs were fabricated using hot embossing and sintering techniques [33]. As demonstrated in Fig. 4b, a mixture of stainless steel powder, paraffin wax as binder, and N,N’-ethylenebis(stearamide) as pore fillers were embossed with a PDMS female mould. In the hot embossing process, the utilisation of the optimum temperature of 95 ± 2 °C and pressure of 1.0 ± 0.1 MPa were investigated, and the MNs were successfully filled in the mould without elastic deformation. Prior to sintering, the binder and pore fillers were decomposed by temperature and dissolution after peeling off the array from the mould. Finally, the MN array was sintered at a temperature of 1100 °C for 30 min, with a final pore size range of 1.56–2.93 µm in the structure and internal porosity of 36 ± 0.37%.

Porous alumina MNs can also be fabricated using the micro-moulding and sintering methods (Fig. 4c). It was demonstrated that the size of the alumina particle, type and amount of porogen, binder, and solvent, and sistering temperature have an impact on the formation of porous structures [66, 69]. The concentrations of different binders such as poly (vinyl alcohol) (PVA) and PVB, which could be dissolved in water and ethanol, respectively, were optimised to effectively fill the micro-mould and produce crack-free and smooth MNs. Consequently, porous alumina structures with pore diameters of up to 1–1.5 µm and porosity of up to 60% were attained. In addition to the size and amount of alumina particles and porogen, the sintering temperature is an important factor in the formation of pores. When sintered at a high temperature (e.g. 1500 °C), particle diffusion and bond formation between particles occurred, and thus a portion of the pores closed or disappeared.

In summary, the sintering process is advantageous for industrial mass production, but it consists of sequential processes and requires specific ovens that can reach high temperatures. In addition, several parameters and conditions of manufacturing, such as alumina particle sizes, solvents, and binders, must be optimised to fabricate robust porous MNs, enabling fast ISF extraction and efficient drug loading.

#### Porogen leaching

The porogen leaching method was proposed to create polymeric porous MNs by washing away the porogen from the body of the MN. One of its advantages in the aspect of fabrication is that the pore size and porosity are both controllable based on the type and amount of used porogen.

**Fig. 5:**
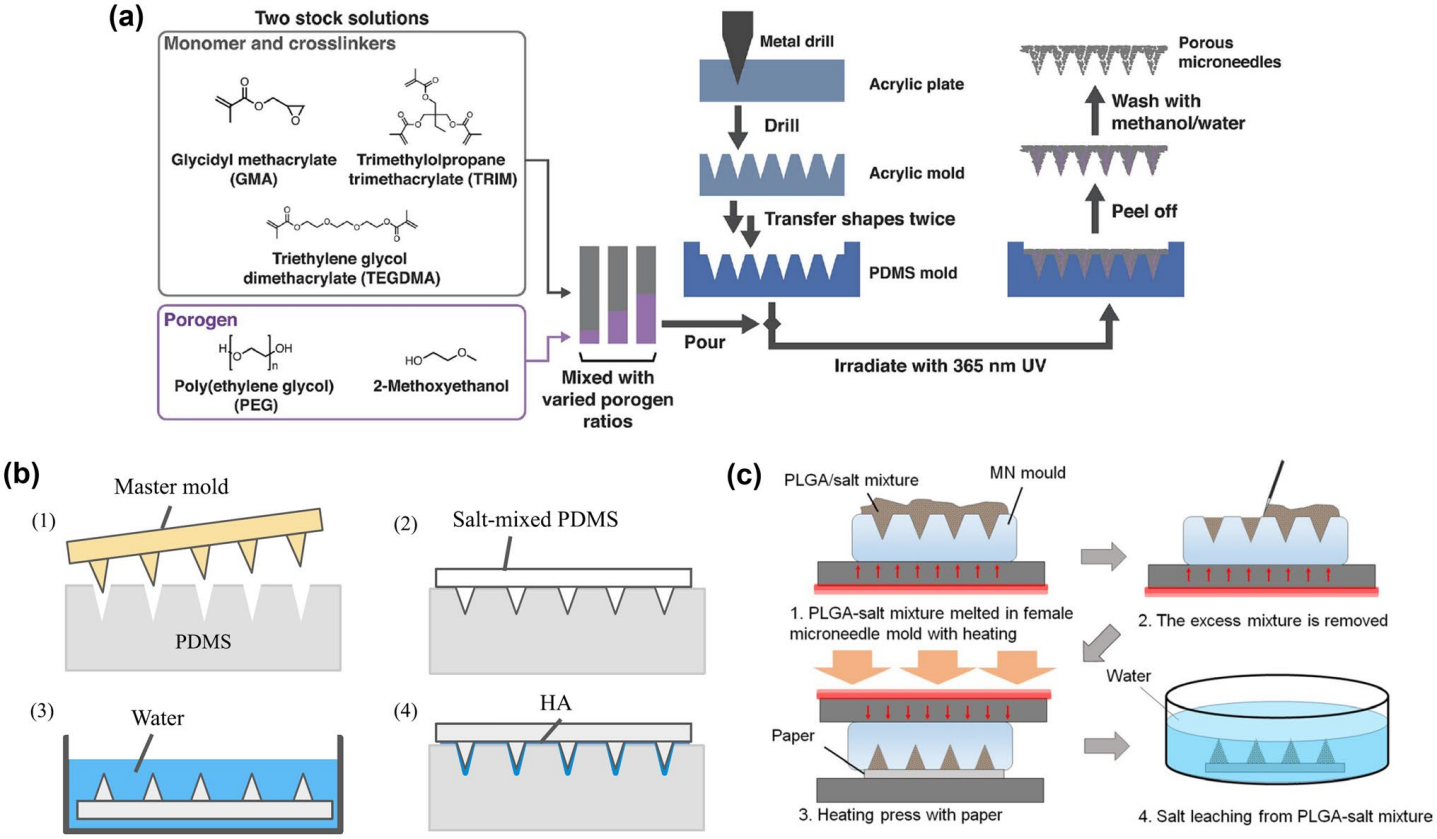
Porogen leaching process to produce continuous pores in MNs: **a** monomer and crosslinkers mixed with poly (ethylene glycol) porogens leached by methanol/water after irradiation with ultraviolet light (reproduced with permission [29]); **b** polydimethylsiloxane (PDMS) blended with salt eliminated by deionised (DI) water after curing (reproduced with permission [34]); **c** poly (lactic-co-glycolic acid) (PLGA)-mixed salt removed by DI water after PLGA solidification (reproduced with permission [58])

PGMA MNs can be synthesised by mixing GMA as monomers and TRIM and TEGDMA as crosslinkers followed by UV irradiation [29]. As a porogen agent, a solution of PEG dissolved in 2-methoxyethanol has been used. Therefore, a PGMA MN array was formed by pouring a mixture of the polymer solution composed of porogens, monomers, and crosslinkers to reticulate by UV irradiation (Fig. 5a). Following PEG porogen leaching and elimination using methanol/water (1:1 volume) resulted in continuous pores in the MNs. Pore sizes of up to 1 µm and the desired porosity and fluid transport speed were obtained by adjusting the porogen ratio of the entire mixture (porogen and monomer solutions). Consequently, with a porosity lower than 50%, more than 80% of the penetration efficiency could be achieved using porcine skin. Conversely, it is difficult to form continuous pores with a low porogen ratio because of the lack of porogen for interconnection, while a high porogen ratio results in blunt tips and fragile structures, which results in low penetration efficiency.

To simplify the number of polymers used and the blending process, PDMS, with salt as a porogen, was proposed for the formation of porous structures [34, 91]. As demonstrated in Fig. 5b, a mixture of PDMS and salt at a volume ratio of 20% was cast into a chemically treated PDMS female mould and cured [34]. After peeling off, the salt in the cured PDMS MN array was leached for 48 h in deionised (DI) water to form the desired porous structures. After the complete removal of salt particles, interconnected voids with diameters of 30–60 µm were formed. However, it was confirmed that the formed MN array shrunk owing to the inherent elastic property of PDMS. In addition, the porous structure turned into sponge-like structures, which resulted in porous MNs with insufficient mechanical strength to penetrate the skin. To solve this issue, dissoluble HA was coated on the porous PDMS MN array to increase the mechanical strength of PDMS MNs.

Similar to PDMS, biodegradable porous PLGA MNs can also be produced using the salt-leaching method [58]. As depicted in Fig. 5c, PLGA powder and salt particles at a pre-determined volume ratio were coarsely mixed and heated at 250 °C to fuse PLGA. The melted PLGA and salt particles were centrifugated several times to blend homogeneously and then cast from a PDMS female mould for an MN array. After cooling down and peeling off, the PLGA/salt mixture-based MN array was immersed in DI water for 24 h to leach the salt from the MN body, which resulted in the formation of PLGA porous MNs with the complete removal of the salt from the MNs. Here, the porosity of PLGA MNs could be tuned by modulating the volume ratio of PLGA and the salt porogen. Moreover, the results indicated that PLGA MNs with 65% porosity and 5–15 µm pore size extracted the largest volume of sample fluids owing to their interconnected pores inside the MN body.

Meanwhile, a porogen-loaded emulsion solution can be coated onto metallic solid MNs and leached out to form porous structures on the surface of MNs [33]. A PLGA dissolved in dichloromethane (DCM) and a PVA solution with gelatine as a porogen were prepared and mixed homogeneously to form an emulsion-based coating solution. Prior to coating, the surface of the stainless steel MN was treated with oxygen plasma and submerged into a polyethyleneimine solution to promote the adhesion of the emulsion. After coating with the emulsion, the MNs were placed at 37 °C for several hours to remove the gelatine, allowing pores to be formed on the surface of the MN. Because the porogen was gelatine, the porosity and pore diameter could be tuned by tweaking the concentration of gelatine in the PVA solution, resulting in the corresponding values of 45–63% and 1.9–6.1 µm.

The porogen leaching method is advantageous because of the simplicity in the modulation of porosity, pore size, mechanical strength, and fluid transport speed; however, the time for the leaching process is relatively long. In addition, proper MN base materials are required to maintain sufficient mechanical strength after the removal of the porogen.

#### Hot embossing

Hot embossing is used to bond polymeric powders inside the body of the MNs so that bonded powders can form interconnected pores [38]. First, a cavity array mould was prepared by drilling aluminium sheets using a laser. Then, PLGA powder was placed on the aluminium mould and slowly pressed at 800 N by the top heating block. The top heating block was set and maintained at a temperature of 65 °C, which was marginally higher than the melting point of PLGA, while the temperature of the bottom block was set to 50 °C, which was below the PLGA melting temperature. Here, PLGA powder was only bonded to the MN body, while it was melted from the GPMA substrate. Consequently, a structure with a gradient of porosity of approximately 20.1% was created, enabling the drug to be stored in the MN body instead of the entire PLGA MN patch for effective drug delivery.

#### Phase separation

**Fig. 6:**
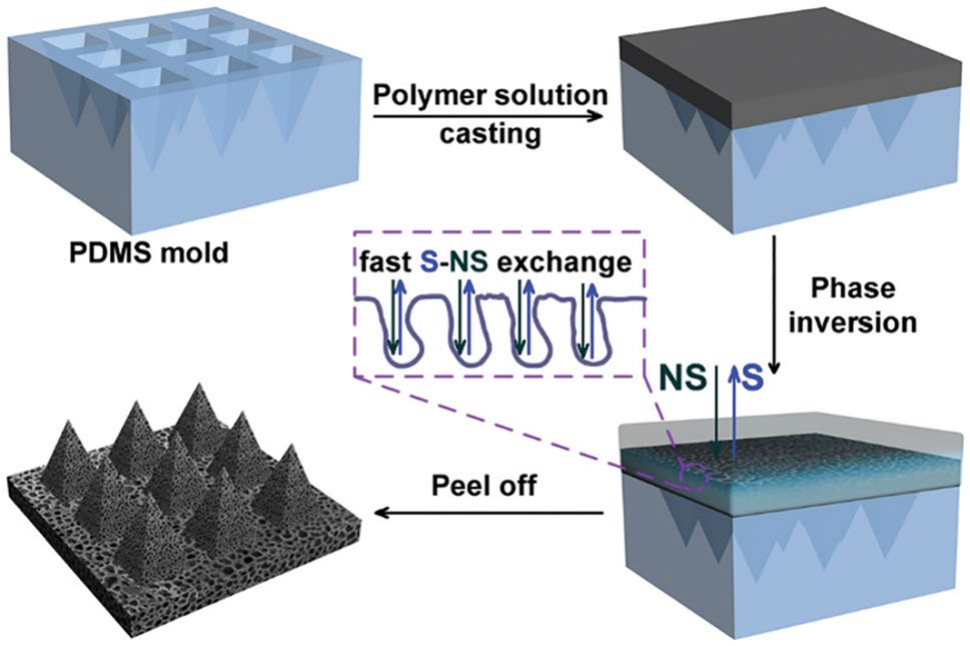
Schematic illustration of phase inversion for forming porous structures, where S and NS refer to solvent and non-solvent, respectively (reproduced with permission [92])

Phase separation was initially aimed at separating two immiscible liquids, creating two distinct phases. During phase separation, the polymer solution is transformed into a solid state when immersed in a non-solvent solution, and pores are formed in the solidified polymers [81, 92, 93]. Certain commonly used polymers, such as cellulose acetate, were successfully applied to the fabrication of continuous pores in the MN body using organic and water phase separation methods as shown in Fig. 6 [92]. First, 35 wt% of cellulose acetate was homogeneously dissolved in DMSO as an organic solvent. As depicted in Fig. 6, the viscous polymer solution was cast onto the prepared PDMS female mould and filled into the cavities through sonication. Then, phase separation was induced by immersing the PDMS mould with a cellulose acetate solution loaded into the water. A porous structure was finally formed during the phase inversion of the DMSO organic solvent in water, and a freeze-drying process was performed to remove the non-solvent portion. The porosity was measured at 45.8% and could be tuned from 40 to 90% by regulating the initial concentration of cellulose acetate in the organic solvent. The phase separation method is an easy and versatile method to fabricate porous MNs using a broad spectrum of polymers. However, more investigations regarding polymer concentration, time for phase inversion, and removal process of the organic solvent are necessary for optimising the fabrication of porous MNs.

#### Emulsion and bonding

The emulsion technique can produce biodegradable microspheres that can be filled into an MN mould to form porous structures [96, 97].

**Fig. 7:**
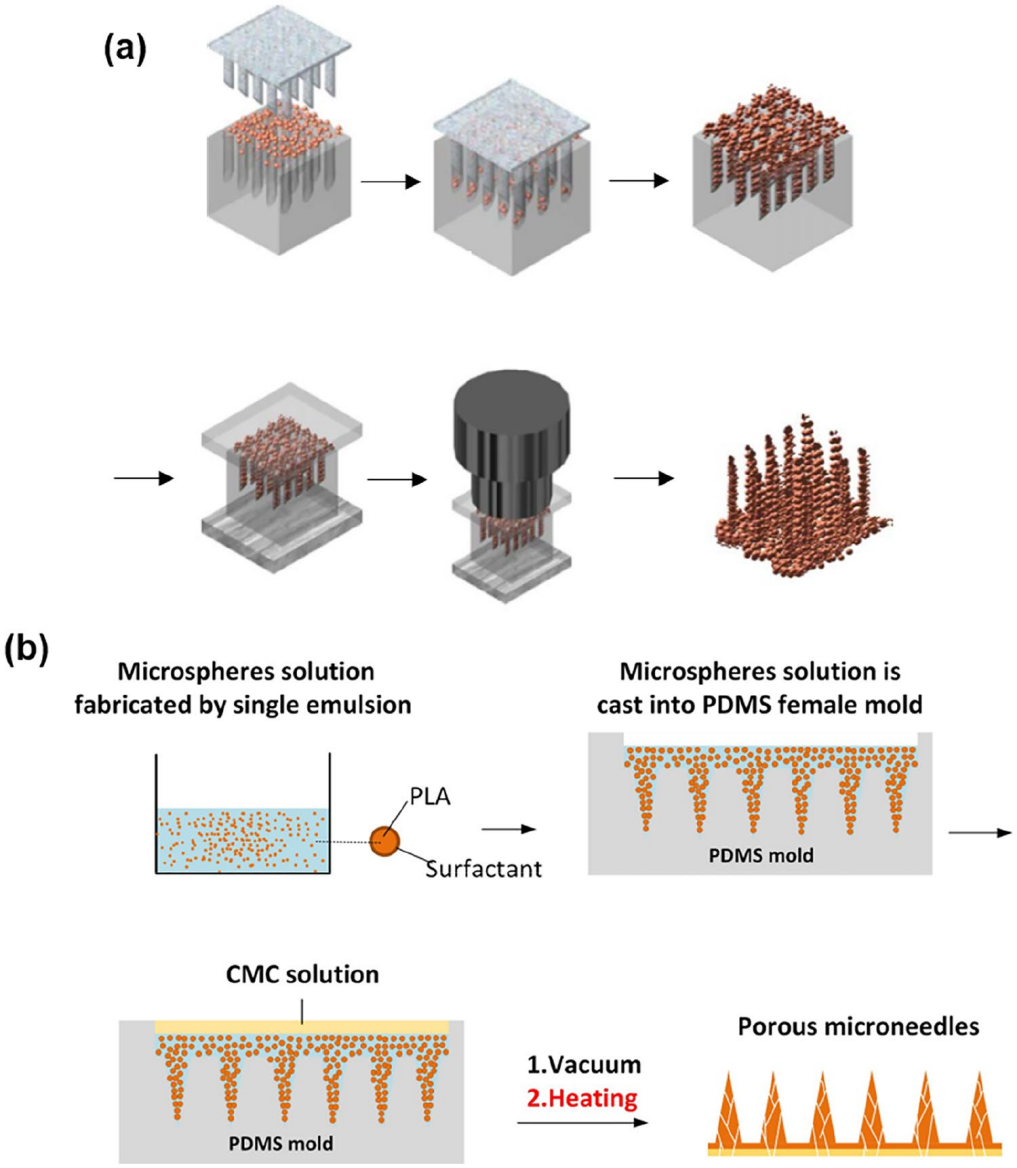
Fabrication methods of porous structures using poly (lactic acid) microparticles to bond together: **a** ultrasonic welding (reproduced with permission [96]); **b** heat treatment (reproduced from [97])

As one of the commonly used biodegradable polymers, PLA has been employed to fabricate microspheres. PLA microparticles with calcein, as an encapsulated model drug, were fabricated by the double-emulsion method [96]. Briefly, a water-in-oil emulsion was obtained by homogenising calcein solution and PLA in DCM solution through a stirring process. Subsequently, the resulting emulsion was homogenised in 0.1% PVA in DI water, which is a surfactant solution, to form a water-in-oil-in-water double emulsion. Then, the emulsion solution was agitated to extract DCM by evaporation. From the emulsion process, PLA microspheres encapsulating calcein were produced and isolated by filtration to yield microparticles with sizes ranging from 1 to 30 µm. The overall fabrication process of the porous PLA MNs is shown in Fig. 7a. First, the prepared PLA microspheres were cast in a PDMS female mould and pushed to fill the cavities by a PDMS male structure. The casting and pushing processes were repeated until the mould was filled with microspheres. A metal plate was then placed at the bottom of the mould while a PDMS sheet covered the top of the mould. Finally, the tip of an ultrasonic horn was pressed against the PDMS mould, and ultrasonic welding was conducted to generate frictional heating between the microspheres, resulting in their bonding. Consequently, interconnected pores in the PLA MNs were directly formed, and the porosity was measured as 75%.

To reduce the complex fabrication procedures and make strong porous structures, heat treatment was proposed as a method to fuse the biodegradable microparticles and bond them together during the micro-moulding process [97]. PLA microspheres can be easily prepared from oil-in-water emulsions using the solvent evaporation method. In brief, PLA powder was dissolved in DCM to prepare the oil phase, followed by the phase where it is mixed into water with PVA as a surfactant. The system was then agitated at room temperature until the DCM fully evaporated, yielding PLA microspheres with an average size of 30 µm. Subsequently, the PLA microsphere solution was poured into a PDMS female mould, followed by centrifugation and vacuum processing to fill the cavities with the microspheres, as demonstrated in Fig. 7b. In addition, carboxymethylcellulose solution was added as the substrate material for the final MN array. The mould was placed in a convection oven and heated at 200 °C, above the melting point of PLA, to fuse and bond them together, resulting in the formation of continuous micropores.

## Biomedical applications of porous MNs

### Drug and vaccine delivery

By taking advantage of the porous structures of MN arrays, either a liquid or a dried drug can be loaded into the MN body and even the backplate as a reservoir. These unique benefits can be achieved only by using porous MNs. For solid and hollow MNs, their APIs are limited to liquid-state drug formulations, while coated and dissoluble MNs use APIs only in dried form. In the case of porous MNs, drugs in the liquid state are introduced and loaded into the pores of the MN body and diffused into the skin after the insertion of MNs. Similarly, the drug in the solid state can be stored in the interconnected pores first. After MN insertion into the skin, the dried drug can be rehydrated by dermal ISF absorbed through capillary action, allowing the drug to diffuse into the skin.

**Fig. 8:**
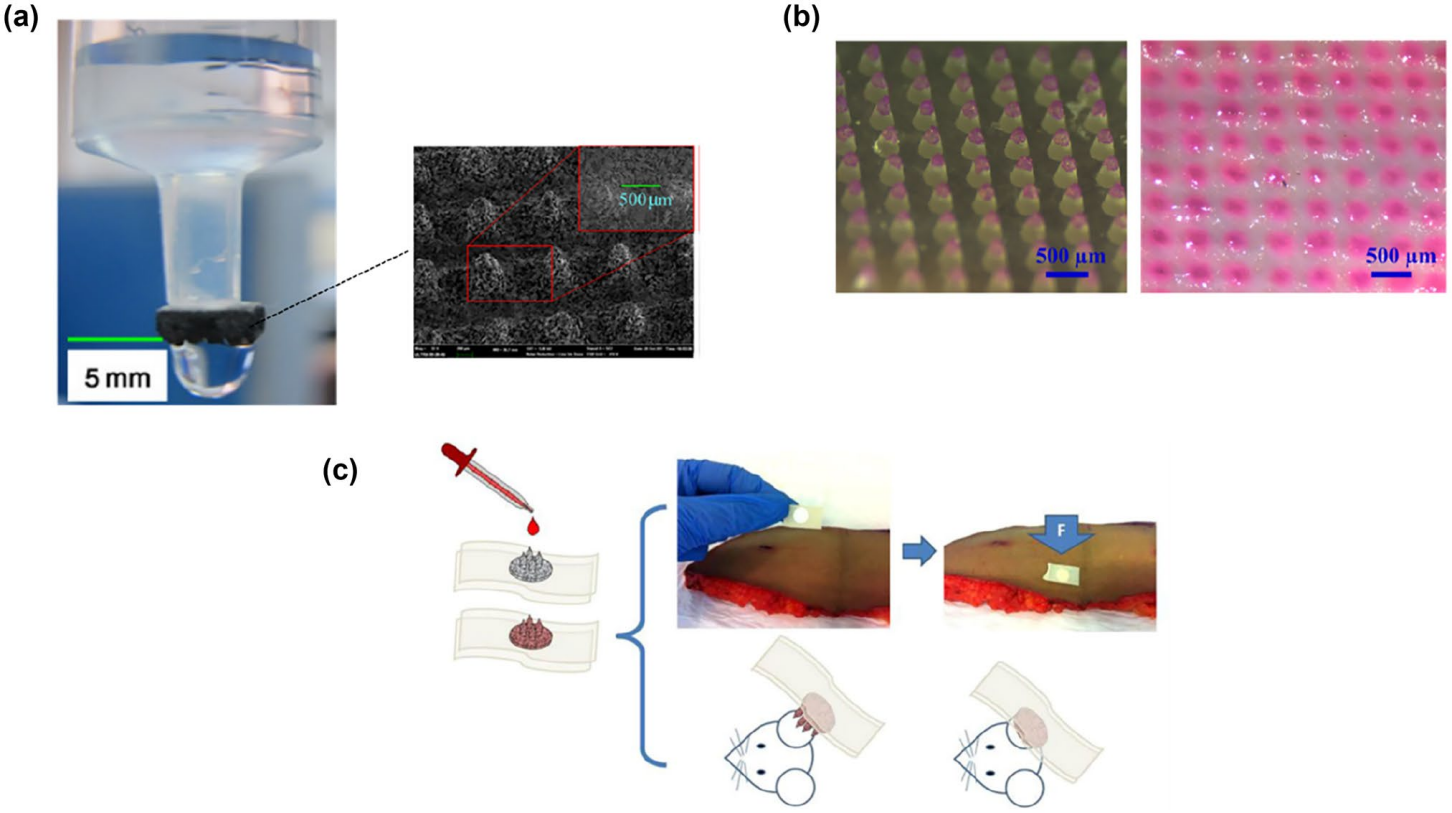
Transdermal drug and vaccine delivery through porous MNs: **a** insulin-loaded syringe connected to porous titanium MNs for painless insulin injection (reproduced with permission [77]); **b** model drug loaded and dried at the tips of gradient porous PLGA MNs and drug diffused inside skin after puncture (reproduced with permission [38]); **c** model vaccine loaded into porous ceramic MNs and applied for transdermal vaccine delivery (reproduced with permission [67])

Proteins such as insulin can be delivered through porous MNs. Diabetic patients are required to inject insulin using hypodermic needles several times a day, which causes repetitive pain to patients at every injection. As an alternative method, an insulin-loaded syringe was connected to the back substrate of a titanium MN array that has entirely porous structures (Fig. 8a) [76]. In this MN device, insulin flowed through the interconnected pores and was delivered into the skin of a rat after the insertion of MNs. The results of in vivo tests demonstrated that porous titanium MNs have the same effect in lowering the blood glucose, showing significant potential in replacing conventional hypodermic injections as well as significantly improving patient compliance. In addition to metals, microporous alumina MNs with tuneable porosity were used for insulin delivery [66]. In particular, the porous alumina MNs revealed the fastest insulin permeation of 62% of the total insulin, which subsequently achieved 80% of the total insulin release within 4.5 h in vitro using rat skin when the solvent of alumina (38 wt%) slurry was ethanol (61 wt%).

Along with non-polymeric MNs, polymer porous MNs have been also used to deliver insulin with the advantage that broken MNs inside the skin degraded over a period of time and eventually disappear [38]. The PLGA porous MNs with gradient porosity were fabricated by loading 5 international units (IU) of insulin, primarily in the tip (Fig. 8b). Then, the effect of drug delivery was compared with a 5 IU insulin injection through in vivo tests using rabbits. The results indicated that insulin-loaded gradient PLGA-based porous MNs slowly and effectively lowered the blood glucose level of rabbits in comparison with the hypodermic injection of insulin at the same dose, which showed the feasibility of insulin delivery with minimal invasiveness using porous MNs.

Cellulose acetate porous MNs with good biocompatibility were investigated for transdermal insulin delivery [92]. The porous cellulose acetate MNs were fabricated and loaded with 10 IU of insulin. The effect of insulin release was then evaluated in vivo by measuring the blood glucose level. The results demonstrated that the blood glucose level of mice decreased when measured 1 h after the application of insulin-loaded porous MNs and dropped to the lowest level after 4 h, showing a promising perspective in transdermal drug delivery.

Similar to drug delivery applications, porous MN arrays can also be applied for vaccine delivery [31, 67, 68]. A dense network of dendritic cells is distributed in human skin, which performs a vital role in inducing the activation of naive CD8^+^ T cells in the immune process against infections and tumours. A nano-porous ceramic MN array with a reservoir in its base plate was loaded with vaccine formulations including peptide antigens followed by a subsequent administration on the ear of a rat through in vivo experiments (Fig. 8c). The results of immunisation demonstrated that effector CD8^+^ T cells were generated and functioned to lyse intracellular pathogens and tumours, which were comparable to vaccine injection using conventional needle injections. Thus, by exploiting the porous structures of MNs, pain-free, sustained, and targeted vaccine delivery can be achieved. Furthermore, porous alumina MNs were used to load and release small molecules and nanoparticles of up to 100 nm in vitro, showing their significant potential for controlled and long-term drug delivery using drug or vaccine-encapsulated nanoparticles.

### Interstitial fluid extraction and subsequent biosensing

ISF in human skin possesses abundant biomarkers and biological information that have been studied and investigated recently as a potential analyte for biomarker monitoring. However, ISF sampling techniques such as suction blister, microdialysis, and reverse iontophoresis approaches in previous research may cause skin damage and require bulky instruments as well as trained medical professionals [56, 98]. Porous MNs can passively absorb ISF through continuous pores by capillary action, which reveals significant potential in painless ISF sampling and additional biosensing as well as in disease diagnosis. Although the continuous voids may increase the fragility of the MN body, porous MNs composed of both non-polymer materials and polymers using various fabrication methods have shown sufficient mechanical strength for successfully piercing skin models or animal skins. For example, stainless steel MNs with interconnected porosity could penetrate skin-mimicking agarose gel (1.4%) with 1 mg/ml of rhodamine B. In addition, the results showed that the metallic MNs could rapidly absorb 20–25 µl of sample fluid in 20 s [33].

Porous alumina MN arrays were also applied for the transdermal extraction of glucose [66]. For the evaluation, agarose gels containing different concentrations of glucose were prepared mimicking the human skin. The porous MNs were then inserted into the agarose gel and held for 15 min. After the extraction process, centrifugation was conducted for the recovery of extracted glucose, which was followed by the measurement of glucose concentration.

In another study, porous MNs made of 35 wt% cellulose acetate were used to extract glucose in vivo [92]. The fabricated patch penetrated the mouse skin resulting in extraction of 1.33 ± 0.11 mg of skin ISF within 10 min, which is sufficient for the measurement of glucose. Here, the extracted ISF had to be recovered by centrifugation and then tested using a glucose assay kit as described earlier. The results showed that the glucose concentrations of extracted ISF were very close to the blood glucose level detected by the commercial glucose meter, indicating that the painless porous MNs can be a proper alternative to measure glucose levels without blood sampling.

**Fig. 9:**
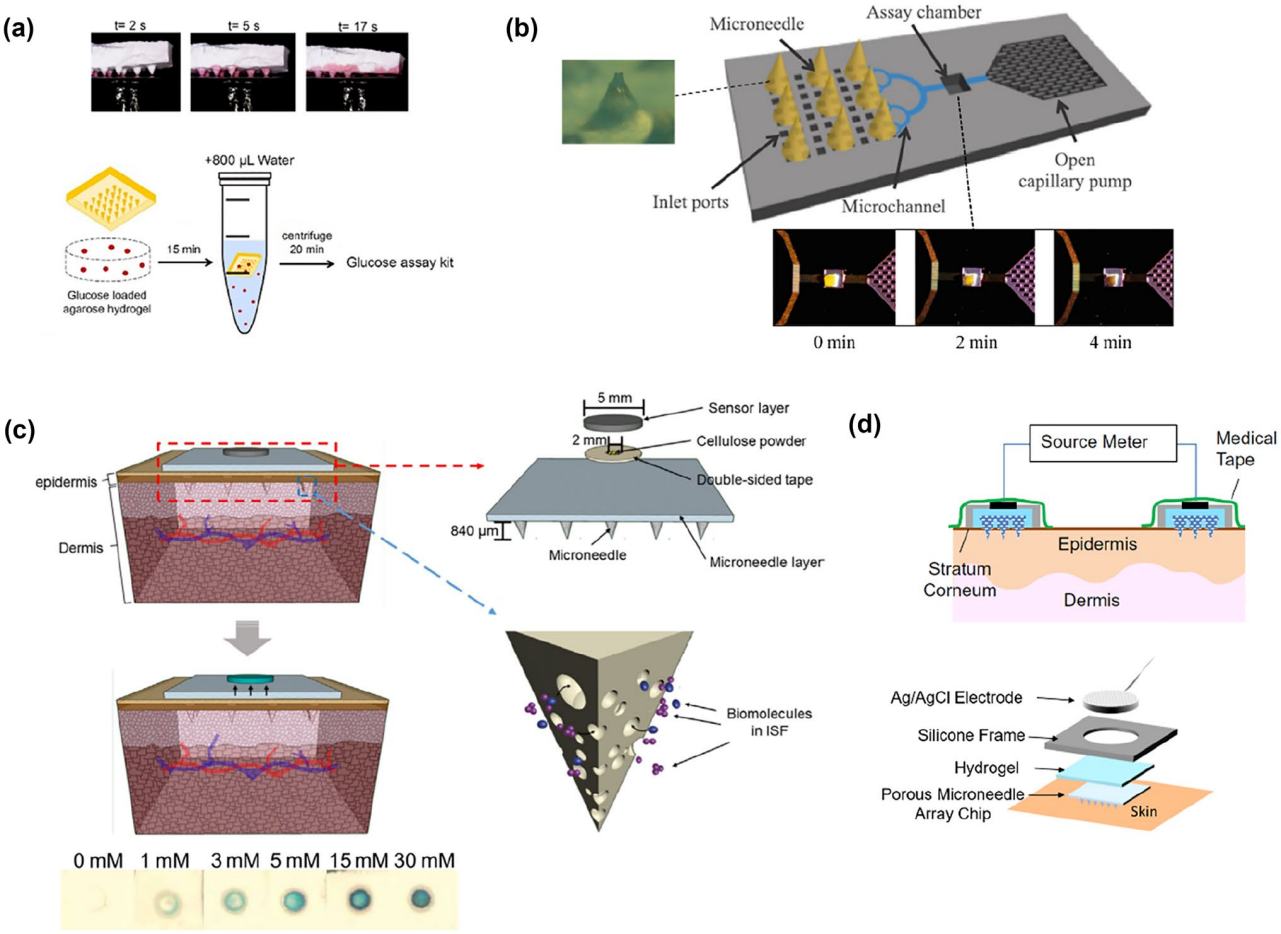
Interstitial fluid extraction and biosensing through porous MNs devices: **a** porous alumina MNs applied for transdermal extraction of glucose which is recovered by centrifugation for subsequent detection (reproduced with permission [66]); **b** porous PDMS MNs interfaced with microfluidic chip for fluids collection and transport, as well as glucose monitoring system in assay chamber (reproduced with permission [34]); **c** porous PLGA MNs loaded on the paper substrate with a paper-based sensor attached for glues concentration test based on the colorimetric analysis of the reaction zone (reproduced with permission [58]); **d** porous poly(glycidyl methacrylate) MNs integrated with electrode system for transdermal electrical diagnosis (reproduced with permission [86])

As the absorption and extraction of the ISF of the skin are the primary functionalities of porous MNs, the combination with a microfluidic chip can simply achieve ISF collection in one device [34]. As demonstrated in Fig. 9a, a microfluidic chip was interfaced with HA-coated porous PDMS MNs, enabling the MN-absorbed fluids to flow through the microchannels for collection in the assay chamber. Subsequent analyses, such as the measurement of glucose concentration, can be achieved by integrating a glucose biosensor in the assay chamber. However, one downside of the integrated microfluidic chip is that users are required to wait 10 min for the HA to be dissolved in the skin after insertion. In addition, manual compression must be repeated approximately 50 times for the permeation of the sample fluid in interconnected pores.

Meanwhile, a porous MN array on a paper substrate integrated with a glucose sensor was proposed for the rapid screening and analysis of glucose concentration. Among several materials for MNs, PLGA was used for fabricating porous structures using the salt-leaching method [58]. The literature showed that porous PLGA MNs possessed sufficient mechanical strength to pierce through the agarose gel-based skin model covered with aluminium foil. In addition, the PLGA MNs with 65% porosity extracted glucose-contained sample fluids and transported the said sample to a paper sensor layer (Fig. 9b), which resulted in a colour change to blue due to dye oxidation resulting from the consumption of glucose by enzymes. Therefore, using the developed patch-type biosensing system with a biodegradable MN array and integrated paper-based sensor layer, the glucose level in ISF can be clearly observed based on the changed colour of the paper sensor. From the results described above, it is expected that the minimally invasive, simple, and integrated MN device can be useful as point-of-care (POC) diagnosis chips as well as home healthcare devices to prevent prediabetes and diabetes.

Meanwhile, local continuous monitoring of intercellular swelling can be performed using a porous MN-based electrode system [86]. PGMA MN arrays produced by crosslinking and porogen leaching methods were used to form porous structures [29]. Ag/AgCl electrodes were placed on the reverse side of the MN substrate, as depicted in Fig. 9c. A source meter was used to connect two MN electrodes, and the DC electrical resistance could be obtained. While the ISF content was applied to DC resistance, interstitial swelling (oedema) was precisely monitored and diagnosed by the pair of porous MN arrays with electrodes. When the observed subject lowered his leg, a reduction in DC resistance was observed as the interstitial water content increased and vice versa. Consequently, it was shown that monitoring and diagnosis of various electrical parameters can be achieved by composing porous MNs and electrodes.

### Prospects and challenges

Porous MNs have a dense network of interconnected capillary channels that enable the absorption of liquids by capillary action, showing a significantly different working principle in comparison with other types of MNs (e.g. solid, coated, hollow, dissoluble, swellable MNs). Thus, either drug loading or ISF extraction can be achieved by using porous MNs arrays. In addition, a wide range of materials, including non-polymers and polymers, can be chosen and used to fabricate porous structures for versatile applications.

While considering drug delivery, solid and hollow MNs can only deliver liquid drug formulations, while drugs in the solid state are delivered by coated and dissoluble MNs owing to their inherent structure and used matrix materials [10]. Conversely, porous MN arrays show more versatility because either liquid or dried drugs can be loaded and stored in the interconnected pores. Liquid drugs can be loaded through capillary action by dipping porous MNs into a drug reservoir, and solid drugs can be immobilised in the porous structure after evaporation of the solvent [68]. Hence, wide choices of drugs and vaccines are applicable for a minimally invasive transdermal delivery using porous MNs.

Furthermore, it can be envisioned that porous MNs offering painless skin penetration and targeting the skin ISF by capillary action can replace existing painful blood sampling methods [26, 34, 58]. In terms of ISF extraction and biosensing, hollow and swellable MNs were widely applied in previous research. However, hollow MNs made of non-biocompatible materials such as silicon or metals could be harmful if broken in the skin [15, 19]. For swellable MNs, ISF absorbed by the swollen hydrogel must be recovered by centrifugation and/or solvent extraction for subsequent biochemical analysis [26, 27]. While considering the availability of materials for hollow and swellable MNs, porous MNs offer more flexibility in material selection and integration with biosensing platforms (e.g. microchip, colorimetry, and electrochemistry) [34, 58, 86]. Furthermore, among the materials usable for fabricating said needles, a wide range of biocompatible and biodegradable materials that were previously investigated can be selected.

However, owing to the inherent elastic property of human skin, the length of MNs that can be inserted into the skin is limited, which must be optimised [30]. Moreover, the mechanical strength of porous MNs is considered to be weaker than that of solid-type MNs because of the continuous voids inside the MN bodies. To overcome this issue, while considering the porous MNs for drug delivery, MNs with porous tips were developed to strengthen the mechanical property, and dose-sparing drug delivery can be easily achieved [38, 61, 70]. Another versatile approach that can enhance the mechanical strength is to coat the porous polymer layer on solid MNs [32]. As for ISF extraction from the human skin, the porosity can be adjusted to enhance the mechanical strength of porous MNs [29, 58].

Recently, the increasing population and rising incidence of chronic diseases such as diabetes and hypercholesterolemia have driven the rapid growth of the POC diagnostic devices market [99]. With these testing kits, increased regular home care and self-testing for early detection of diseases can be achieved. Currently, blood sampling (e.g. through a finger-prick method) is still the predominant method for biomolecular collection [100]. However, regular usage of such sampling methods necessarily results in patients bearing a significant amount of pain repetitively when a metallic needle pierces the skin to reach the blood vessels. Therefore, there is an urgent requirement for developing minimally invasive and simple-to-use POC devices to improve the compliance of users or patients, which can promote their preventive action and increase the frequency of monitoring.

Although certain technical breakthroughs have been achieved, the industrialisation and commercialisation of porous MN devices have still been impeded for the following reasons. The current porous MN patches are designed to be small in size for primary utilisation through in vivo experimental tests. Thus, large-sized MN patches should be considered for practical applications in the real world related to human beings. Moreover, the substrate of the porous MN patch should be thin and flexible to be attached to the human skin. In addition, several reported fabrication processes suitable for laboratory scale might not be transferable to the industrial scale for mass production. In particular, the relatively high cost of the matrix material and manufacturing could limit the scale-up in the industry. Furthermore, the complicated and time-consuming fabrications that require several steps might increase the cost while diminishing the efficiency of production. Further, when assembled with biosensors for early diagnosis, affordable price and adequate reliability should also be considered for a competitive advantage in the current POC devices market. In addition, several years are required to design MN-based sensing devices for commercialisation due to the regulations that require the evaluation of accuracy and reliability through long-term clinical tests. Moreover, the sterilisation, usage, and disposal should be standardised for consumers or patients to handle medical devices appropriately and safely at home. Nevertheless, there is no doubt that in the near future, MN-based healthcare POC devices would be commercialised and even integrated with wireless communication modules to achieve comprehensive personal healthcare, which will result in the rapid growth of the POC market aimed at home health devices.

## Conclusion

This review primarily focused on and described porous MNs. The different characteristics of several other types of MNs were summarised and compared in terms of their inherent structures and working principles. In particular, for interconnected porous MNs, the continuous nano- or micro-sized pores offer a driving force for fluid transportation by capillary action. A wide range of matrix materials from non-polymers to polymers, which can be selected to fabricate a porous matrix, were listed and described. Various fabrication methods for porous structures were extensively developed based on the materials used, of which the processes and their advantages and disadvantages were also summarised. It is significantly important to optimise porous MNs with sufficient mechanical strength to maintain the MN structures for successful penetration into the skin of the patient. Moreover, fabrication methods of porous MNs that are suitable for scaling up mass production still require investigation and development. Porous MNs show promising perspectives in drug/vaccine delivery (e.g. small molecules, nanoparticles, insulin, peptide vaccines) as well as ISF extraction/biosensing (e.g. microfluidic chip, colorimetry, electrochemistry). In particular, it can be envisioned that portable and minimally invasive POC devices comprising porous MNs and miniaturised biosensors for early and rapid diagnosis of diseases would perform a major and crucial role in home healthcare for the increase of self-testing and preventive action.

## Data Availability

Not applicable.
